# Supplementary material for: WordCommentsAnalyzer: A windows software tool for qualitative research
Source: F1000Res. 2018 Sep 4;7:536. Originally published 2018 May 3. [Version 2] doi: 10.12688/f1000research.14819.2 (PMC6137411; doi:10.12688/f1000research.14819.2)
Supplement: Supplementary file 3 [file f1000research-7-17614-s0002.tgz › b2a79496-4ada-47c4-9682-a99ce867836d.zip!/Eight Iranian Health Journals/Yafteh _27.docx]

© Yafteh  . This work is licensed under CC BY 4.0 (https://creativecommons.org/licenses/by/4.0/).

Volume & Issue: دوره 14 - شماره 1

Title: تأثیر GVHD مزمن بر بقا بیماران لوسمی میلوئیدی حاد پس از پیوند سلول‌های بنیادی خون محیطی از خواهر یا برادر با HLA مشابه بدون تخلیه سلول T

http://yafte.lums.ac.ir/article-1-702-fa.html

مقدمه: بیماری پیوند علیه میزبان (GVHD) یکی از معضلات اصلی پیوند سلول‌های بنیادی خونساز آلوژنیک است و همچنین یکی از فاکتورهای مهم مؤثر بر نتیجه پیوند می‌باشد. یک شیوع افزایش یافته GVHD متعاقب پیوند سلول‌های بنیادی خون محیطی (PBSC) آلوژنیک مطرح شده است، اگرچه، چگونگی تأثیر این مورد بر روی بقا هنوز به خوبی مشخص نشده است. در این مطالعه، هدف ما ارزیابی تأثیر GVHD حاد (aGVHD) و GVHD مزمن (cGVHD) بر روی بقا کلی(OS) ، بقا بدون بیماری (DFS) و عود متعاقب پیوند سلول‌های بنیادی خون محیطی (PBSCT) از خواهر یا برادر با HLA مشابه بدون تخلیه سلول T بود. مواد و روش‌ها: اطلاعات حاصل از 78 بیمار، شامل 40 بیمار با لوسمی میلوئیدی حاد (AML) و 38 بیمار با لوسمی لنفوبلاستیک حاد (ALL)، تحت PBSCT آلوژنیک از خواهر یا برادر با HLA مشابه بدون تخلیه سلول T آنالیز شدند. تمام بیماران یک رژیم یکسان آماده-سازی ریشه‌کن کننده مغزاستخوان و پیشگیری کننده GVHD را دریافت کرده بودند. ما شیوع aGVHD و cGVHD و تأثیر آنها بر روی بقا و عود در این بیماران را بررسی کردیم. بحث و نتیجه‌گیری: این نتایج نشان می دهد که وقوع cGVHD یک پیشگویی کننده مهم نتیجه PBSCT آلوژنیک از خواهر یا برادر با HLA مشابه بدون تخلیه سلول T است که در آن بیماران AML که cGVHD را گسترش می‌دهند شانس بقا بیشتری دارند. یافته‌ها: شیوع کلی aGVHD و cGVHD، 5/82٪ و 5/42٪ در بیماران AML و 2/84٪ و 3/26٪ در بیماران ALL بود. وقوع GVHD حاد تأثیری بر روی OS، DFS و عود در بیماران AML و ALL دریافت کننده پیوند نداشت. اگرچه شیوع OS و DFS دو ساله در بیماران AML با GVHD مزمن در مقایسه با بیماران بدون GVHD مزمن به‌طور معنی‌داری بالاتر بود (به ترتیب 024/0= Pو 033/0=P)، ولی این اختلاف ناشی از کاهش شیوع عود نبود.

Title: بررسی کیفیت خدمات بهداشتی درمانی ارائه شده در مراکز بهداشتی درمانی شهر خرم آباد با استفاده از مدل سروکوال در سال 1389

http://yafte.lums.ac.ir/article-1-703-fa.html

مقدمه: به منظور ارائه بهتر خدمات در مراکز بهداشتی درمانی، آگاهی از ادراک و انتظارات دریافت‌کنندگان خدمات این مراکز و تعیین شکاف موجود بین این دو مقوله نقش بسیار مهمی را ایفا می‌کند. این مطالعه با هدف ارزیابی کیفیت خدمات بهداشتی درمانی ارائه شده در مراکز بهداشتی درمانی شهر خرم آباد با استفاده از مدل سروکوال در سال 1389 انجام شد. بحث و نتیجه‌گیری: انتظارات مراجعین در تمام ابعاد کیفیت ارائه خدمات بالاتر از ادراکات آن‌ها بود و نیاز است کیفیت ارائه خدمات در این مراکز در تمام ابعاد به ویژه بعد همدلی بهبود یابد. پیشنهاد می‌شود کیفیت ارائه خدمات در این مراکز به صورت دوره‌ای بررسی و به منظور بهبود ارائه خدمات بهداشتی درمانی اقدامات لازم صورت گیرد. یافته‌ها: در تمامی ابعاد پنج‌گانه کیفیت خدمات ارائه شده (شامل ابعاد ملموس یا فیزیکی، قابلیت اطمینان، پاسخگویی، تضمین، و همدلی) شکاف منفی کیفیت وجود داشت.‌ بیشترین میانگین شکاف کیفیت در بعد همدلی و کمترین میانگین شکاف مربوط به بعد قابلیت اطمینان بود. بین سن مراجعین و نمره شکاف کیفیت همبستگی معکوسی وجود داشت(026/0=p). رابطه آماری معنی‌داری بین شکاف کیفیت با جنس و تحصیلات مراجعین مشاهده نشد. مواد و روش‌ها: در این مطالعه توصیفی- تحلیلی مقطعی، 650 نفر از مراجعین به مراکز بهداشتی درمانی شهر خرم‌آباد با استفاده از روش نمونه‌گیری طبقه‌ای و تصادفی انتخاب شده و مورد بررسی قرار گرفتند. جمع‌آوری داده‌ها با استفاده از پرسشنامه استاندارد سروکوال انجام شد. تجزیه و تحلیل داده‌ها با استفاده از نرم افزار SPSS 16 و به کمک آمار توصیفی و آزمون‌های ویلکاکسون، من‌ویتنی، کروسکال والیس و ضریب همبستگی اسپیرمن انجام گرفت.

Title: بررسی میزان شیوع انتریت عفونی ناشی از کمپیلوباکترجوجنای در کودکان مبتلا به اسهال شهرستان خرم‌آباد

http://yafte.lums.ac.ir/article-1-704-fa.html

مقدمه: اسهال کودکان یکی از معضلات عمده‌‌ بهداشتی مردم دنیا به ‌خصوص کشورهای در حال توسعه می‌باشد که هر سال حدود 4 تا 5 میلیون کودک به دلیل این عارضه جان خود را از دست می‌دهند. کمپیلوباکتر جوجنای یکی از باکتری‌های بیماری‌زای شایع انسانی می‌باشد که حداقل برابر سالمونلا و شیگلا به عنوان عامل اسهال‌های عفونی نقش دارد. با توجه به مطالعات محدود انجام شده در رابطه با جنبه‌های مختلف اپیدمیولوژیک و شناختی این باکتری در ایران، پژوهش حاضر با هدف تعیین انتریت عفونی ناشی از کمپیلوباکتر جوجنای در کودکان مبتلا به اسهال شهر خرم‌آباد انجام گرفت. مواد و روش‌ها: این تحقیق به منظور بررسی توصیفی اسهال‌های ناشی از کمپیلوباکتر جوجنای در کودکان زیر 12 سال شهر خرم‌‌آباد از شهریور سال 1385 تا شهریور سال 1386 انجام گرفت. نمونه‌ها، از مدفوع کودکان مبتلا به اسهال حاد برداشت و بر روی محیط انتقالی به آزمایشگاه میکروبیولوژی آموزشکده‌ دامپزشکی دانشگاه لرستان انتقال ‌یافت و سپس جهت جداسازی و تشخیص کمپیلوباکتر جوجنای کشت داده شدند. بحث و نتیجه‌گیری: بر اساس نتایج این مطالعه، شیوع انتریت کمپیلوباکتری در کودکان زیر 12 سال شهرستان خرم‌آباد در سال 1386-1385، 92/5 درصد بوده است که با نتایج گزارش شده از سایر مطالعات انجام شده در ایران از جمله زاهدان (6 درصد)، تهران (5 درصد)، ساری (8/4 درصد) و شیراز (5 درصد) مشابهت دارد. یافته‌ها: از تعداد کل 321 نمونه‌ جمع‌آوری شده، 130 نمونه (5/40 درصد) مربوط به کودکان دختر(جنس مؤنث) و 191 نمونه (5/59 درصد) مربوط به کودکان پسر(جنس مذکر) بود. تعداد کودکان مبتلا به انتریت کمپیلوباکتری 19 مورد (92/5 درصد) بود که شامل 11 مورد (43/3 درصد) از نمونه‌های دختر و 8 مورد (49/2 درصد) از نمونه‌های پسر بود. بیشترین موارد مثبت در گروه‌های سنی زیر یک سال (87/1 درصد) و یک تا سه سال (87/1 درصد) بود که این تفاوت بر حسب گروه‌های سنی مورد مطالعه از نظر آماری معنی‌دار بود.

Title: همه‌گیرشناسی پرده بکارت در مراجعین به واحد معاینات اداره کل پزشکی قانونی فارس

http://yafte.lums.ac.ir/article-1-706-fa.html

مقدمه: معاینه تخصصی پرده بکارت آثار متعدد فردی، خانوادگی، اجتماعی و قانونی به همراه دارد؛ لذا شناخت همه گیرشناسی آن برای پزشکان و ماماها ضروری است. مواد و روش‌ها: در یک مطالعه مقطعی - تحلیلی زنان مراجعه کننده به بخش معاینات زنان مرکز پزشکی قانونی شیراز در طی یک سال از نظر سلامت یا ازاله، زمان حدوث پارگی، نوع صدمه فیزیکی، محل آناتومیک، علت مراجعه و سایر موارد معاینه تخصصی شدند. بحث و نتیجه‌گیری: شکل تشریحی پرده بکارت تنوع فراوانی داشته و افتراق یافته های طبیعی از یافته های غیرطبیعی در معاینه بکارت اهمیت خاص خود را دارد. یافته‌ها: در مجموع 823 نمونه و با میانگین سنی 98/2± 78/21 سال و با بیشترین فراوانی رده سنی 20-16 سال وارد مطالعه گردیدند. 58/53% پرده‌ها غیرقابل اتساع و 17/46% پرده‌ها قابل اتساع و دو مورد بدون منفذ بودند. از نظر نوع پرده، بیشترین فراوانی برابر 99/86 درصد پرده حلقوی با لبه صاف و بیشترین فراوانی زمان حدوث ازاله بکارت برابر 23/80 درصد بیش از سه هفته قبل از مراجعه بود. میان وضعیت پرده بکارت از نظر سلامت یا ازاله و گروه های سنی مراجعین و هم چنین میان سن مراجعین و زمان حدوث ازاله پرده بکارت رابطه معنی دار(001/0=p)مشاهده گردید.

Title: بررسی فراوانی آنمی فقر آهن و عوامل مرتبط با آن در بیماران بالغ مراجعه کننده به کلینیک داخلی بیمارستان شهدای عشایر شهرستان خرم‌آباد از اسفند 1389لغایت تیرماه1390

http://yafte.lums.ac.ir/article-1-705-fa.html

مقدمه: آنمی یکی از مشکلات شایع جهان و ایران است و همچنین آنمی فقر آهن شایع‌ترین مشکل کم خونی تغذیه ای در دنیا است. مطالعه زیر با توجه به شیوع بالای آنمی فقر آهن و وابستگی شدت آن با سطح فرهنگی-اقتصادی هر منطقه برای دستیابی به آمار شیوع و برنامه‌ریزی و درمان، طراحی و اجرا گردیده است. بحث و نتیجه‌گیری: با توجه به شیوع بالای آنمی فقر آهن در جامعه مورد پژوهش، توجه به مداخلات ارتقایی و پیشگیرانه و برنامه‌های آموزشی جامع در این زمینه ضروری به نظر می‌رسد. یافته‌ها: از کل افراد مبتلا به آنمی شرکت کننده در مطالعه 4/44% بیماران مبتلا به آنمی فقر آهن بودند. شیوع آنمی فقر آهن در زنان 3/52% و در مردان 6/22% بود. 9/60% افراد 15-49 سال و5/24% افراد بالای 49 سال مبتلا به آنمی فقر آهن بودند. بین سن و جنس و آنمی فقر آهن ارتباط آماری معنی‌داری دیده شد(004/0=p). بین سابقه مصرف داروهای ضد التهاب غیر استروییدی (NSAIDs) و بروز آنمی فقر آهن ارتباط آماری معنی‌داری دیده نشد(33/0=p). مواد و روش‌ها: این مطالعه به صورت مقطعی و با روش سرشماری صورت گرفته است.کلیه بیماران بالغی که با علائم و نشانه های آنمی به کلینیک داخلی بیمارستان شهدای عشایر خرم آباد مراجعه کردند، تحت بررسی CBC و ایندکس‌های RBC قرار گرفتند.سپس همه افرادی که میزان هموگلوبین خون آن‌ها در محدوده تعریف آنمی بود به عنوان نمونه پژوهش وارد مطالعه شدند. برای تمامی این بیماران سنجش سطح آهن، فریتین سرم وTIBC صورت گرفت و پرسش نامه ای برای هرکدام تکمیل شد. سپس نتایج از نظر آماری بررسی شد.

Title: بررسی اثر کورکومین بر سطح پروتئین های AQP1 در سلول های اپیتلیالی شبکه کوروئیدی بطن‌های جانبی رت نژاد ویستار

http://yafte.lums.ac.ir/article-1-707-fa.html

مقدمه: کانال‌های پروتئینی آکواپورین1 (AQP1) موجود در سلول‌های شبکه کوروئیدی نقش مهمی در تولید مایع مغزی- نخاعی (CSF) ایفا می‌کنند. بیماری‌هایی نظیر افزایش فشار داخل جمجمه‌ای، هایپوناترمیای سیستمیک و هیدروسفالی با افزایش CSF همراه هستند. مطالعات نشان می‌دهند که کورکومین توانایی مهار کانال‌های یونی را دارد. لذا هدف این تحقیق، بررسی توانایی کورکومین برای کاهش بیان پروتئین‌های AQP1 در سلول‌های شبکه کوروئیدی می‌باشد. مواد و روش‌ها: در این مطالعه سلول‌های شبکه کوروئیدی از بطن‌های جانبی مغز رت استخراج شده و در محیطDMEM کشت داده شدند. پس از یک هفته کورکومین در دوزهای μM20 وμM30 طی 24 ساعت و دوزهایμM 10 وμM15 طی 48 ساعت بر روی سلول‌ها تأثیر داده شد. تأثیر کورکومین بر میزان بقاء این سلول‌ها با سنجشMTT و اثر آن بر تغییر سطح پروتئین AQP1 با روش فلوسیتومتری بررسی شد. بحث و نتیجه‌گیری: با توجه به این بررسی کورکومین می‌تواند بیان AQP1 را در شبکه کوروئید کاهش دهد و به عنوان یک داروی گیاهی ممکن است بتواند با کاهش دادن میزان تولید CSF در درمان بیماری‌هایی نظیر افزایش فشار داخل جمجمه‌ای، هایپوناترمیای سیستمیک و هیدروسفالی مفید باشد. یافته‌ها: نتایج حاصل از سنجشMTT میزان IC50کورکومین را بر روی این سلول‌ها پس از 24 و 48 ساعت به ترتیب μM40 و μM22 نشان داد. نتایج حاصل از فلوسیتومتری نشان داد که کورکومین به طور وابسته به دوز و زمان تا حدود 50% باعث کاهش بیان AQP1 در این سلول‌ها می‌شود.

Title: نتایج اجرای مداخلات پیشگیری ثانویه بیماری‌های قلبی عروقی در طرح مداخلاتی جامعه‌نگر برنامه قلب سالم اصفهان

http://yafte.lums.ac.ir/article-1-710-fa.html

مقدمه: طرح زندگی سالم برای بیماران قلبی (HLCP) یکی از ده طرح برنامه قلب سالم اصفهان (IHHP) بود که با توجه به استراتژی‌های پیشگیری ثانویه طراحی و اجرا شد. هدف از اجرای این پروژه کاهش وقایع مجدد قلبی و نیز عوامل خطر، ناخوشی و مرگ و میر در بیماران مبتلا به بیماری قلبی عروقی (CVD) بود. در این مطالعه به ارزشیابی فرایند اجرای این طرح و بررسی اثر اجرای آن بر مصرف داروهای پیشگیری ثانویه در مبتلایان به CVD در طول مدت مطالعه پرداختیم. بحث و نتیجه‌گیری: نتایج به دست آمده نشان دهنده این است که می‌توان با استفاده از امکانات موجود مداخلات پیشگیری ثانویه را در مبتلایان به CVD اجرا کرد. یافته‌ها: از بین مداخلات اجرا شده، آموزش بیماران در زمان بستری، زمان ترخیص و پس از آن از مداخلات موفق بودند. به علاوه جلب همکاری مردمی با همکاری یک تشکل غیردولتی از موفقیت‌های این طرح در ادغام برنامه‌های پیشگیری ثانویه در سطح جامعه بود. علاوه بر این مصرف داروهای آسپرین، بتابلوکرها، مهار کننده‌های آنزیم مبدل آنژیوتانسین Angiotensin Converting Enzyme Inhibitor(ACE-I) و استاتین‌ها در طول مطالعه در مبتلایان به CVD افزایش معنی‌دار داشت. مواد و روش‌ها: در این طرح پنج استراتژی اصلی برای دستیابی به اهداف ذکر شده طراحی شد. مداخلات طرح بر اساس آموزش، قانون‌گذاری و جلب همکاری‌های بین بخشی و مردمی طراحی شد. برای بررسی اجرای این مداخلات و میزان موفقیت آن‌ها، ارزشیابی فرایند انجام گرفت. به علاوه پیش از آغاز مداخلات وضعیت موجود مصرف داروها در مبتلایان به CVD بررسی گردید.

Title: بررسی رابطه سبک رهبری مدیران و فرسودگی شغلی کارکنان ستادی دانشگاه علوم پزشکی لرستان در سال 1390

http://yafte.lums.ac.ir/article-1-711-fa.html

مقدمه: از آنجا که شاغلان بیش از 60 درصد زمان بیداری خود را در محیط کار سپری می‌کنند، این محیط نقش مهمی در ارتقای سلامت افراد ایفا می‌کند. امروزه در بسیاری از سازمان‌ها، فشارهای عصبی و روانی به درجات گوناگونی وجود دارد. یکی از پیامدهای فوری استرس و فشارهای روانی، پدیده فرسودگی شغلی است . از طرفی یکی از مهمترین عوامل مولد فرسودگی شغلی در سازمان ، سبک رهبری خاصی است که توسط سرپرستان سازمان اتخاذ می گردد. مواد و روش‌ها: این پژوهش یک مطالعه توصیفی- همبستگی است که با هدف بررسی رابطه سبک رهبری مدیران با فرسودگی شغلی کارکنان ستادی دانشگاه علوم پزشکی لرستان انجام گرفت. روش نمونه گیری در این پژوهش تصادفی- طبقه ای بوده و حجم نمونه بر اساس جدول مورگان تعیین گردیدکه شامل 182 نفر از کارکنان و 45 نفر از مدیران می باشد . ابزار گردآوری داده ها ، شامل دو پرسشنامه استاندارد بوده است. 1- پرسشنامه سبک رهبری فیدلر(LPC) 2- پرسشنامه فرسودگی شغلی مسلش. اعتبار پرسشنامه ها از طریق ضریب آلفای کرونباخ برای پرسشنامه فرسودگی شغلی 84% و برای پرسشنامه سبک رهبری 81% محاسبه گردید. برای تجزیه وتحلیل داده های آماری از نرم افزار SPSS استفاده گردید. بحث و نتیجه‌گیری: نتایج این پژوهش نشان داد که بین سبک رهبری مدیران و فرسودگی شغلی کارکنان با اطمینان بالای 99% رابطه معکوس معنی دار وجود دارد ،به عبارت دیگر فرسودگی شغلی بیشتر قربانیان خود را از بین سازمان‌ ها و مؤسساتی می‌گیرد که رفتا‌ر های ملاحظه کارانه و گرم و عاطفی کمتری از طرف مدیران و رؤسای آنها سر می‌زند. وجود ارتباط بین سبک رهبری مدیران و فرسودگی شغلی کارکنان ، توجه هر چه بیشتر مدیران به پرسنل و استفاده از شیوه های علمی مدیریت را می طلبد. یافته‌ها: 3/64% کارکنان فرسودگی پایین،2/35 % فرسودگی متوسط و کمتر از یک درصد فرسودگی بالا را نشان داده اند و33/93% از مدیران این دانشگاه در حوزه های ستادی از سبک‌های رهبری وظیفه مدار44/4 % از سبک‌های رابطه مدار و22/2 % از سبک‌های بینابین استفاده می‌نمایند، همچنین بین سبک رهبری مدیران و فرسودگی شغلی کارکنان دانشگاه علوم پزشکی لرستان رابطه معنی‌دار وجود دارد.

Title: تأثیر تاب‌آوری بر سلامت روانی و رضایت از زندگی، یک الگوی روان‌شناختی از بهزیستی

http://yafte.lums.ac.ir/article-1-712-fa.html

مقدمه: رویکرد روان‌شناسی مثبت‌گرا تبلور تغییر در علم روان‌شناسی از صرف پرداختن به ترمیم آسیب‌ها به سمت بهینه‌سازی کیفیات زندگی است. این جنبش در پی آن است که از نقاط قوت انسان‌ها به عنوان سپری در مقابل بیماری روانی بهره گیرد. در این میان تاب‌آوری به عنوان یکی از زیرطبقات این رویکرد نقش مهمی در مقابله با تنیدگی‌ها و تهدیدهای زندگی و آثار نامطلوب آن دارد؛ به شکلی که در طی یک دهه اخیر جایگاه ویژه‌ای را در حوزه بهداشت روان به خود اختصاص داده است. پژوهش حاضر با هدف بررسی تأثیر تاب‌آوری بر سلامت روانی و رضایت از زندگی انجام شد. مواد و روش‌ها: در بررسی مقطعی 178 نفر (86 زن و 92 مرد) از شاغلین به تدریس در مراکز آموزش و پرورش شهرستان رامشیر ارزیابی شدند برای گردآوری داده‌ها مقیاس تاب‌آوری (CD-RIS)، مقیاس سلامت عمومی (GHQ-28)، مقیاس رضایت از زندگی (SWLS) و پرسش‌نامه ویژگی‌های جمعیت‌شناختی به کار برده شد. برای تحلیل داده‌ها از رگرسیون چندگانه به روش سلسله مراتبی هم زمان استفاده شد. بحث و نتیجه‌گیری: تاب‌آوری به واسطه افزایش سطح سلامت روانی، رضایت از زندگی را در پی دارد. هم‌چنین متغیر تاب‌آوری دارای اثر غیرمستقیم بر رضایت از زندگی است. به بیان دیگر تاب آوری با تأثیر بر نوع احساس‌ها و هیجان‌های فرد، نگرش مثبت و در نتیجه رضایت از زندگی را در پی دارد. یافته‌ها: نتایج رگرسیون چندگانه به روش سلسله مراتبی هم زمان، بیانگر معنی‌داری نقش واسطه‌ای سلامت روان، در رابطه میان تاب آوری (001/0>p) و رضایت از زندگی (001/0>p) بود. هم‌چنین یافته‌های پژوهش حاضر بیانگر تأثیر تاب‌آوری در افزایش سلامت روانی و رضایت از زندگی بود. میانگین سنی آزمودنی‌ها 16/32 سال (انحراف معیار70/5) بود.

Title: مقایسه بازشناسی بیان چهره‌ای هیجان در بیماران مبتلا به افسردگی اساسی و افراد بهنجار

http://yafte.lums.ac.ir/article-1-713-fa.html

مقدمه: از جمله عوامل هیجانی دخیل در اختلال افسردگی اساسی، بازشناسی بیان چهره‌ای هیجان است. هدف از پژوهش حاضر مقایسه بازشناسی بیان چهره‌ای هیجان در بیماران مبتلا به افسردگی اساسی و افراد بهنجار بود. بحث و نتیجه‌گیری: نقص در بازشناسی بیان چهره‌ای هیجان سازه‌ای است که در تشدید علائم افسردگی اساسی نقش بسزایی دارد. یافته‌ها: از لحاظ بازشناسی بیان چهره‌ای هیجان بین افراد بهنجار (68/2= SD، 95/26= M) و افسرده (97/3= SD، 65/22= M) تفاوت معنی‌داری وجود دارد (05/0>P)، به طوری‌که افراد بهنجار از لحاظ متغیر فوق در وضعیت بهتری از افراد مبتلا به افسردگی اساسی قرار داشتند. مواد و روش‌ها: بیست بیمار مبتلا به افسردگی اساسی و بیست نفر از افراد بهنجار به روش نمونه گیری در دسترس انتخاب شدند و به آزمون بازشناسی بیان چهره‌ای هیجان اکمن و فرایسن (1976) با پایایی 71/0، پاسخ دادند. روش پژوهش، مورد‌‌- شاهدی بوده و برای تحلیل داده‌ها از تحلیل واریانس چند متغیره (مانووا) استفاده شده است.

Title: مقایسه دو روش هموستاز در میزان خونریزی حین عمل جراحی بافت نرم اندام فوقانی

http://yafte.lums.ac.ir/article-1-714-fa.html

مقدمه: توقف خونریزی طی عمل جراحی نیازمند هموستاز به صورت مکانیکی ، گرمایی یا شیمیایی است . هدف از این پژوهش مقایسه دو روش هموستاز در میزان خونریزی حین عمل جراحی بافت نرم اندام فوقانی است . بحث و نتیجه‌گیری: الکتروکوتردارای عوارض نکروز بافتی است در صورت امکان در طی عمل‌های جراحی بافت نرم محدود شود گرچه تآیید نتایج نیازمند تحقیق بیشتر است . یافته‌ها: میانگین سنی گروه هموستاز ترکیبی ( کوترو تورنیکت) 54/8 ±62/31 و گروه هموستاز مکانیکی 05/8 ±9/29 سال بود. دو گروه از نظر سنی تفاوت معنی دارآماری نداشتند (6/0=p) . 8/63 درصد از نمونه ها را مردان و 2/36 درصد را زنان تشکیل داده بودند. بیشترین مورد جراحی مربوط به ترمیم پارگی تاندونهای فلسکور ساعد با 5/37 درصد و کمترین 5/12 درصد مربوط به کیست و جسم خارجی بود. میانگین زمان تورنیکت در گروه آزمون و شاهد به ترتیب 3/4 ±87/27 و 6/6 ±4/ 29 و میانگین حجم خون از دست رفته در گروه آزمون و شاهد به ترتیب 3/5 ±3/16 و 7/4 ±2/ 17 بود . آزمون آماری بیانگر نبود تفاوت معنی دار آماری در دو گروه بود (1/0=p). مواد و روش‌ها: در این مطالعه مورد – شاهدی 80 بیمار دارای معیارهای ورود به مطالعه ( سن کمتر از 50 سال، نداشتن ترومای شکمی، BMI(کمتر از30 ) وآاسیب بافت نرم ) که توسط یک جراح تحت عمل جراحی قرار گرفته بودند با نمونه گیری در دسترس (با در نظر گرفتن معیارهای ورود به مطالعه ، سن و جنس به صورت غیراحتمالی) به دو گروه آزمون و کنترل تقسیم شدندو مداخله در گروه آزمون مبتنی بر برقرای هموستاز با الکتروکوتردر بدو جراحی و سپس بستن تورنیکه بود ولی در گروه کنترل بدون استفاده ازکوتربرای ایجاد هموستاز از بدو عمل جراحی برای بیماران تورنیکت استفاده شد. حجم خون از دست رفته مبتنی بر تعداد گازهای مصرفی بودو در طی عمل از ساکشن برای هیچ کدام از موارد استفاده نشد. داده ها با استفاده از ابزار spss و آمار تحلیلی و توصیفی تجزیه و تحلیل شد.

Title: گزارش یک مورد تراتوم خوش خیم مدیاستن خلفی تقلید کننده کیست هیداتیک و یک مورد تراتوم خوش خیم مدیاستن قدامی، تشخیص و درمان

http://yafte.lums.ac.ir/article-1-715-fa.html

مقدمه: تراتوم ها جزء تومورهای مادرزادی سلول زایا طبقه بندی شده اند. این تومورها، بافت های اکتوپیکی هستند که از 3-2 لایه زاینده ساخته شده و شامل اجزای بالغ، نابالغ یا بدخیم هستند و معمولاً در طول خط وسط بدن یافت می شوند. این تومورها به طور شایع در تخمدان ها، بیضه ها، رتروپریتوئن و ناحیه ساکروکوکسیژئال دیده می شوند. تراتوم های مدیاستن، شیوع بسیار کمی داشته به طوری که فقط % 13-8 تومورهای این ناحیه را شامل می شوند. اکثریت آن ها در مدیاستن قدامی قرار گرفته و فقط %8-3 از مدیاستن خلفی منشأ گرفته اند. معرفی مورد : دو بیمار مبتلا به تراتوم خوش خیم معرفی می شود. مورد اول: مرد 65 ساله با شکایت درد قفسه سینه و دیسفاژی مراجعه نموده است که درآن CT اسکن توده کیستیک خوش خیم بسیار بزرگ در مدیاستن خلفی مشاهده و تحت رزکسیون جراحی قرار گرفت، تشخیص نهایی تراتوم کیستیک گزارش شد. علائم بیمار با رزکسیون جراحی از بین رفت. مورد دوم: خانم 55 ساله با تراتوم خوش خیم مدیاستن قدامی که در بررسی به دنبال ترومای قفسه سینه کشف شد و تحت رزکسیون قرار گرفت و در پیگیری 30 ماهه هیچ کدام از بیماران مشکلی نداشتند. بحث و نتیجه‌گیری: این تومورهای با رشد آهسته اغلب بدون علامت اند و به طور تصادفی در تصویربرداری از قفسه سینه و یا به دلیل ایجاد عوارضی چون آتلکتازی، چسبندگی، فشار به ساختمان های مجاور یا پاره شدن و عفونت ثانویه شناسایی می شوند. عمل جراحی تنها درمان قطعی است و حتی وقتی بیمار بدون علامت است به خاطر عوارض بالقوه اش قابل توصیه است.
